# Flavor improvement of enzymatic oyster juice by sequential heating through the accumulation of free amino acids and volatile flavor compounds

**DOI:** 10.3389/fnut.2026.1907081

**Published:** 2026-07-17

**Authors:** Jing Wang, Tianyu Chen, Yanle Jiang, Chunsheng Li, Jie Hou, Xiaoqing Huang

**Affiliations:** 1 Guangdong Provincial Key Laboratory of Advanced Biofermentation Technology Enterprise in Flavoring & Food, Foshan Haitian (Gaoming) Flavouring & Food Co., Ltd., Foshan, China; 2 Key Laboratory of Aquatic Product Processing, Ministry of Agriculture and Rural Affairs, National R&D Center for Aquatic Product Processing, South China Sea Fisheries Research Institute, Chinese Academy of Fishery Sciences, Guangzhou, China; 3 Fisheries College, Jimei University, Xiamen, China; 4 Foshan Haitian Flavouring & Food Co., Ltd., Foshan, China

**Keywords:** flavor, free amino acids, heating treatment, oyster juice, volatile flavor compounds

## Abstract

Protease hydrolysis for production of oyster juice can greatly increase the nutrients and umami ingredients from oyster, but lacks the unique flavor of traditional oyster sauce. This study evaluated a sequential heating treatment consisting of primary heating at 107 °C for 6 h followed by secondary heating at 120 °C for 4 h and investigated the associated flavor-improvement mechanisms by detection of free amino acids and volatile flavor compounds. Compared to the unheated enzymatic juice, the concentrations of amino acid nitrogen and total free amino acids increased progressively after heating and reached their highest levels after secondary heating reaching 0.37 g/100 g and 3.02 g/100 g, respectively. The taste activity value of free amino acids in the enzymatic oyster juice was markedly promoted after sequential heating, especially some umami and sweet amino acids such as Glu and Ala. A total of 667 volatile flavor compounds were detected by HS-SPME-GC–MS, and 133 key volatile flavor compounds were screened out by OAV ≥ 1, mainly including aldehydes, alcohols, ketones, esters, heterocyclic compounds, and terpenoids. The total concentration of key volatile flavor substances in the enzymatic oyster juice increased significantly after two heating processes, rising from 34.30 mg/kg before heating to 75.51 mg/kg and then to 118.15 mg/kg. The odor profile of enzymatic oyster juice showed that the pleasant volatile flavor such as nutty, almond, fruity, green, and sweet odor was significantly enhanced by sequential heating, while the unpleasant volatile flavor such as fishy, earthy, fatty, sulfury, and spicy odor was significantly weakened. Correlation analysis and differential formation pathways showed that these changes were mainly attributed to thermal reaction such as Maillard reaction and Strecker degradation. Sequential heating is an effective method to obviously improve the flavor of enzymatic oyster juice.

## 1 Introduction

Oysters are one widely consumed aquatic product due to their high nutritional value and characteristic flavor (1). Oysters are often processed into oyster sauce to increase their added value. As the main ingredient of oyster sauce, oyster juice is traditionally produced through boil of whole oyster (2), result in low nutrients and umami ingredients such as free amino acids and umami peptides (3). Compared with the traditional boiling method, protease hydrolysis can promote the release of nutrients and umami substances, particularly free amino acids and peptides, during oyster juice production (4, 5). Nevertheless, the enzymatic oyster juice lacks the distinctive flavor of oyster sauce because of fast protein degradation (3). Therefore, how to improve the flavor of enzymatic oyster juice is an important problem to be solved in the oyster sauce production industry.

Heating is an effective technology to improve the flavor of aquatic products because it can drive the production of flavor compounds by thermal chemical reactions including oxidation of lipids and proteins and Maillard reaction (6, 7). Lipids and proteins through oxidation reactions under high temperature can form various free fatty acids and amino acids, and further produce small molecule volatile flavor compounds such as aldehydes, ketones, alcohols, and esters, endowing the aquatic products with unique smell (8, 9). Moreover, free amino acids can not only directly provide taste for aquatic products, but also act as important precursors to produce volatile flavor compounds through the Maillard reaction (10). Numerous studies have shown that various volatile flavor compounds such as aldehydes, ketones, alcohols, sulfur-containing compounds, and heterocycles are obviously improved after heating treatment, endowing the aquatic products a pleasant aroma (11, 12). Current researches on flavor of oyster juice mainly focuses on enzymatic hydrolysis (13) or simple heating treatment (14). However, the contents of free amino acids and volatile flavor compounds cannot meet the requirements for high-quality oyster sauce production. Sequential heating is regarded as a potential method to improve flavor in foods, because it can promote the production of more flavor compounds by thermal reaction such as Maillard reaction (15). However, there is a lack of research on the flavor enhancement of enzymatic oyster juice through sequential heating treatment, and the mechanisms by which sequential heating improves the flavor of enzymatic oyster juice have not been systematically elucidated.

In this study, sequential heating was used to improve the flavor of enzymatic oyster juice. The changes in amino acids and their taste activity value (TAV) in oyster juice were comparatively analyzed under different heating treatments. The volatile flavor compounds were identified and quantitatively analyzed using HS-SPME-GC–MS, followed by the selection of key volatile flavor compounds according to their odor activity value (OAV). Based on these analyses, the flavor-enhancing effect of sequential heating on enzymatic oyster juice was elucidated. This study provides a theoretical foundation and technical support for the development of precise flavor control strategies and the production of high-quality oyster juice.

## 2 Materials and methods

### 2.1 Materials

The same batch of fresh oysters (*Crassostrea angulata*) were purchased from a local seafood market in Foshan, Guangdong, China, and transported to the laboratory within 1 h in insulated containers with ice packs (0–4 °C). Alkaline protease (600,000 U/g) and flavor protease (150,000 U/g) were obtained from Novozymes (China).

Amino acid standard substances and 3-hexanone-2,2,4,4-d4 (internal standard, ≥99%) were purchased from Sigma-Aldrich (USA). N-hexane (chromatographic grade) was purchased from Merck KGaA (Germany). NaOH, formaldehyde and sodium chloride (analytical grade) were purchased from Sinopharm Chemical Reagent Co., Ltd. (China).

### 2.2 Sequential heating of enzymatic oyster juice

Fresh oyster was obtained by the local seafood market (Foshan, China), and then was shelled, washed, and minced. The samples were then mixed with 0.1% alkaline protease (600,000 U/g) and 0.2% flavor protease (150,000 U/g) for enzymolysis at 50 °C and pH 6.5 for 5 h. After centrifugation at 5000 r/min and 4 °C for 20 min, the supernatant was obtained as the enzymatic oyster juice (CK group). The enzymatic oyster juice was placed into the reactor liner, which was then sealed and assembled into the HT-1000FJ high-pressure reactor (Shanghai Huotong Experimental Instrument Co., Ltd.) to prevent the loss of volatile flavor compounds and ensure uniform heating. The reactor was programmed to first heat at 107 °C for 6 h (H1 group). Subsequently, the temperature increased to 120 °C and was maintained for 4 h (H2 group). The samples were stored at −80 °C before analysis.

### 2.3 Determination of amino acid nitrogens

The detection of amino acid nitrogen was performed according to previous study (2) with some modification. Briefly, the sample (5.0 g) was transferred into a 100 mL volumetric flask and was diluted to 100 mL with distilled water. Subsequently, 20 mL of this dilution was combined with 60 mL distilled water and stirred magnetically while adjusting the pH to 8.2 using standardized NaOH solution. Afterward, 10 mL of 38% formaldehyde was added and mixed thoroughly. The mixture was then further titrated with the same NaOH solution to pH 9.2. The concentration of amino acid nitrogen was calculated according to the Equation 1:

$$X=\frac{(V1-V2)\times 0.051\times 0.014}{5\times \frac{V3}{100}}\times 100$$ (1)

where *X* represents the amino acid nitrogen concentration (g/100 mL), *V*1 represents the volume (mL) of the NaOH standard solution consumed during titration after formaldehyde addition to the diluted sample supernatant, *V*2 represents the corresponding NaOH volume (mL) for the blank prepared by replacing the supernatant with distilled water, and *V*3 represents to the aliquot volume (mL) of the sample dilution used in the assay.

### 2.4 Determination of free amino acids

The quantitative analysis of free amino acids was carried out using the LA8080 amino acid automatic analyzer (Hitachi, Japan) (9). The chromatographic column was a sulfonic acid type cation resin, and the detection wavelengths were 570 nm and 440 nm. The mixed amino acid standard working solution and the sample determination solution were injected into the amino acid analyzer in the same volume, and the amino acid concentrations in the sample determination solution were calculated by the external standard method based on the peak area.

The TAV of free amino acid in the oyster juice was calculated using the Equation 2:

$$\mathrm{TAV}=\frac{C_{a}}{TT_{a}}$$ (2)

where *TAV* represents the taste activity value of free amino acid, *C_a_* represents the concentration of free amino acid (g/100 g), and *TT_a_* represents the taste threshold of free amino acid (g/100 g).

### 2.5 Determination of volatile flavor compounds

The determination of volatile flavor compounds was based on the HS-SPME-GC–MS method used in previous study (16). The sample (0.5 mL) were placed in a 20 mL headspace vial. An internal standard (3-hexanone-2,2,4,4-d4) and 4 mL of saturated sodium chloride solution was added. The headspace vial was immediately sealed. Volatile flavor compounds were extracted using a fully automated headspace solid-phase microextraction (HS-SPME) system coupled to gas chromatography–mass spectrometry (GC–MS). The mixture was shaken for 5 min at a constant temperature of 60 °C. A 120 μm DVB/CWR/PDMS extraction head (Agilent, United States) was inserted into the headspace bottle of the sample, and head-space extraction was carried out by SPME Arrow (CTC Analytics AG, Switzerland) for 15 min. The sample was then desorbed at 250 °C for 5 min, followed by GC–MS separation and identification. The extraction head was aged at 250 °C in the Fiber Conditioning Station for 5 min before sampling. The new extraction head was aged in the Fiber Conditioning Station for 2 h before extraction.

The Agilent 8890-7000E GC–MS system (Agilent, United States) was equipped with capillary column (DB-5MS, 30 m × 0.25 mm × 0.25 μm, Agilent, United States). The carrier gas is high-purity helium (≥ 99.999%), the constant flow rate is 1.2 mL/min, the injection port temperature is 250 °C, and the sample is injected in a non-split mode with a solvent delay of 3.5 min. The temperature program was as follows: 40 °C for 3.5 min, 10 °C /min to 100 °C, 7 °C /min to 180 °C, 25 °C /min to 280 °C holding 5 min. Mass spectrometry was conducted in electron ionization (EI) mode at 70 eV, with ion source, quadrupole, and transfer line temperatures set at 230 °C, 150 °C, and 280 °C, respectively. Selected ion monitoring (SIM) was used for detection.

Each compound was identified based on two criteria: (1) matching retention time (±0.2 min) to standards and (2) the presence of one quantitative ion and ≥ 2 qualitative ions in the sample mass spectrum after background subtraction. The concentration of volatile flavor compounds was determined by internal standard method and the Equation 3:

$$C_{v}=\frac{A_{V}\times M_{i}}{A_{i}\times M_{O}}$$ (3)

where *C_v_* represents the concentration of volatile flavor compound (mg/kg), *A_v_* represents the peak area of each volatile flavor compound, *A_i_* represents the peak area of internal standard, *M_i_* represents the weight of internal standard (μg), and *M_0_* represents the weight of oyster juice (g).

The OAV of volatile flavor compound in the oyster juice was calculated using the Equation 4:

$$\mathrm{OAV}=\frac{C_{v}}{T_{v}}$$ (4)

where *OAV* represents the odor activity value of volatile flavor compound, *C_v_* represents the concentration of volatile flavor compound (mg/kg), and *T_v_* represents the threshold of volatile flavor compound (mg/kg).

### 2.6 Statistical analysis

All experiments were performed in three independent trials, and the data were expressed as mean ± SD. The one-way analysis of variance with Tukey test was used to analyze the difference in concentrations of flavor compounds in different groups by IBM SPSS Statistics 22.0. The similarity of flavor compounds in different groups was analyzed by the principal component analysis (PCA). The bubble chart, grouped scatter plot, and heatmap were drawn by Origin 2022.

## 3 Results

### 3.1 Improvement of free amino acids in enzymatic oyster juice by sequential heating

**Figure 1:**
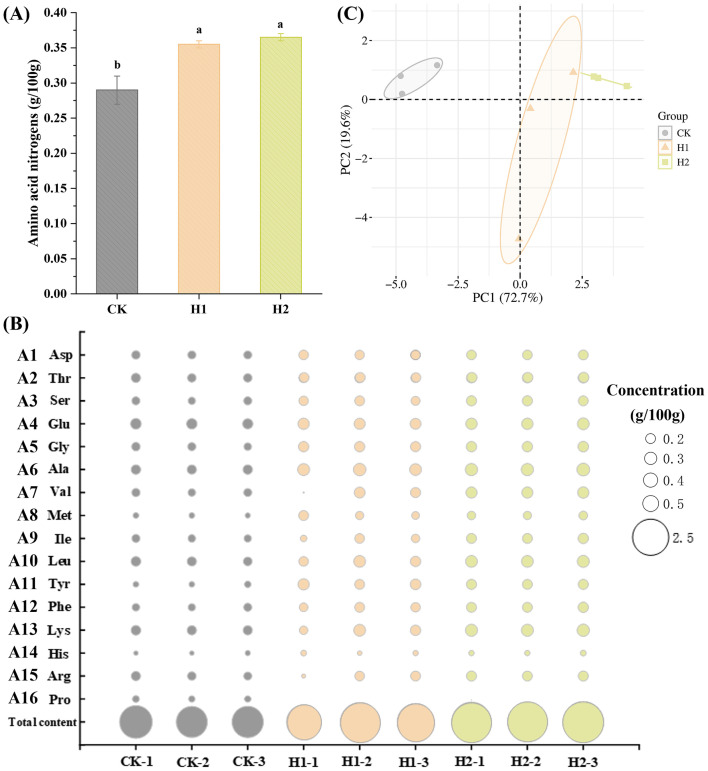
Effect of sequential heating on free amino acids in enzymatic oyster juice. **(A)** Amino acid nitrogen, **(B)** free amino acids, and **(C)** similarity analysis of free amino acids in different treatment groups.

Free amino acids represent key nutritional constituents and major contributors to the taste characteristics of oyster juice (5). The amino acid nitrogen can directly reflect the accumulation of free amino acids in oyster juice. As shown in the Figure 1A, the amino acid nitrogen concentration of oyster juice in the H1 and H2 group was much higher than that in the CK group, and reached the maximum value (0.37 g/100 g) in the H2 group, indicating that sequential heating could induce the formation of more free amino acids. Similarly, the total concentration of free amino acids in oyster juice obviously increased after heating (Figure 1B; Supplementary Table S1), especially the highest content reaching 3.02 g/100 g after secondary heating (H2). The content of Ala was the highest in the process, followed by Glu, Lys, and Leu. The abundant of Ala and Glu has also been observed in the enzymatic oyster juice by alcalase and trypsin (13, 14).

Notably, Ala with sweetness taste increased after heating, and the maximum value appeared in H2 group reaching 0.29 g/100 g. Moreover, other sweetness amino acids such as Ser, Gly, Thr, and Lys also increased markedly after heating. Umami amino acids usually include Glu and Asp., which are the main taste compounds in oyster juice. After heating, the content of Glu and Asp was markedly enhanced, especially in the H2 group reaching 0.24 g/100 g and 0.17 g/100 g. These results suggest that sequential heating is an effective strategy to enhance the sweet and umami taste of the oyster juice, which can be attribute to the thermal degradation of proteins or peptides (1).

The PCA score plot of free amino acids and amino acid nitrogen showed clear separation among the CK, H1, and H2 groups (Figure 1C). The overall variation of PCA reached 92.3% with PC1 and PC2 explaining 72.7 and 19.6% of the total variance, respectively, could well reveal the similarity of free amino acids among different groups. Good parallelism was observed among each parallel sample. The distance between the H1 and H2 groups was relatively close to each other and was more different from the CK group, suggesting that heating treatment had a significant impact on the concentration of free amino acids.

TAV is often used to assess the contribution of a single taste substance to the overall taste of aquatic product. It is generally believed that when the TAV value is greater than 1, this taste substance makes a significant contribution to the taste of the food (9). The TAV of free amino acids in different treatment groups of enzymatic oyster juice is shown in Table 1. The TAV of Glu which showed umami taste was the highest in the enzymatic oyster juice, and significantly increased after heating reaching the maximum value of 8.22 in the H2 group. As the other umami amino acid, the TAV of Asp also significantly increased and reached 1.73 in the H2 group. Ala was the sweet amino acid with the highest TAV, while the sweet amino acids including Gly and Ser only exceeded 1 after heating treatment. These results suggested that sequential heating could enhance the umami and sweet flavor of oyster juice. Besides, the TAV of free amino acids which exhibited both bitter and sweet taste including Val, Met, and His also exceeded 1. Moreover, the TAV of bitter amino acids such as Ile, Leu, Phe, Tyr, and Arg also increased after heating. The thermal effect promoting the bitter taste has also been found in other study because of the release of hydrophobic amino acids (5). Among them, the TAV of Tyr decreased after the second heating compared to the first heating, indicating that sequential heating can slightly eliminate the bitter taste brought by Tyr after the first heating.

**Table 1:** TAV of free amino acids in enzymatic oyster juice in different treatment groups.

Free amino acids	Taste threshold g/100 g	CK	H1	H2
Asp (umami)	0.10	1.27 ± 0.12^b^	1.60 ± 0.09^a^	1.73 ± 0.06^a^
Glu (umami)	0.03	7.00 ± 0.33^b^	7.57 ± 0.21^ab^	8.22 ± 0.38^a^
Thr (sweet)	0.26	0.56 ± 0.04^b^	0.65 ± 0.04^b^	0.74 ± 0.02^a^
Gly (sweet)	0.13	0.95 ± 0.12^b^	1.50 ± 0.04^a^	1.56 ± 0.04^a^
Ala (sweet)	0.06	2.78 ± 0.10^b^	4.63 ± 0.33^a^	4.94 ± 0.19^a^
Ser (sweet)	0.15	0.76 ± 0.08^b^	1.09 ± 0.10^a^	1.24 ± 0.04^a^
Lys (sweet)	1.17	0.14 ± 0.01^b^	0.17 ± 0.06^ab^	0.24 ± 0.00^a^
Pro (sweet)	0.30	0.28 ± 0.02	0	0
Val (bitter/sweet)	0.04	3.00 ± 0.25^a^	3.43 ± 2.95^a^	5.83 ± 0.14^a^
Met (bitter/sweet)	0.03	1.89 ± 0.19^b^	4.57 ± 1.29^a^	4.33 ± 0.33^a^
His (bitter/sweet)	0.02	2.17 ± 0.29^a^	2.50 ± 0.50^a^	2.67 ± 0.29^a^
Ile (bitter)	0.09	1.22 ± 0.11^b^	1.41 ± 0.50^ab^	2.07 ± 0.13^a^
Leu (bitter)	0.19	0.86 ± 0.08^b^	1.11 ± 0.24^ab^	1.44 ± 0.03^a^
Phe (bitter)	0.09	1.22 ± 0.11^b^	1.77 ± 0.24^a^	2.07 ± 0.13^a^
Tyr (bitter)	0.073	0.92 ± 0.16^b^	2.89 ± 0.41^a^	2.55 ± 0.07^a^
Arg (bitter)	0.05	2.80 ± 0.40^a^	2.53 ± 1.69^a^	3.73 ± 0.12^a^

The different lowercase letters in same row indicate the significant difference among groups (*p* < 0.05).

### 3.2 Improvement of volatile flavor compounds in enzymatic oyster juice by sequential heating

**Figure 2:**
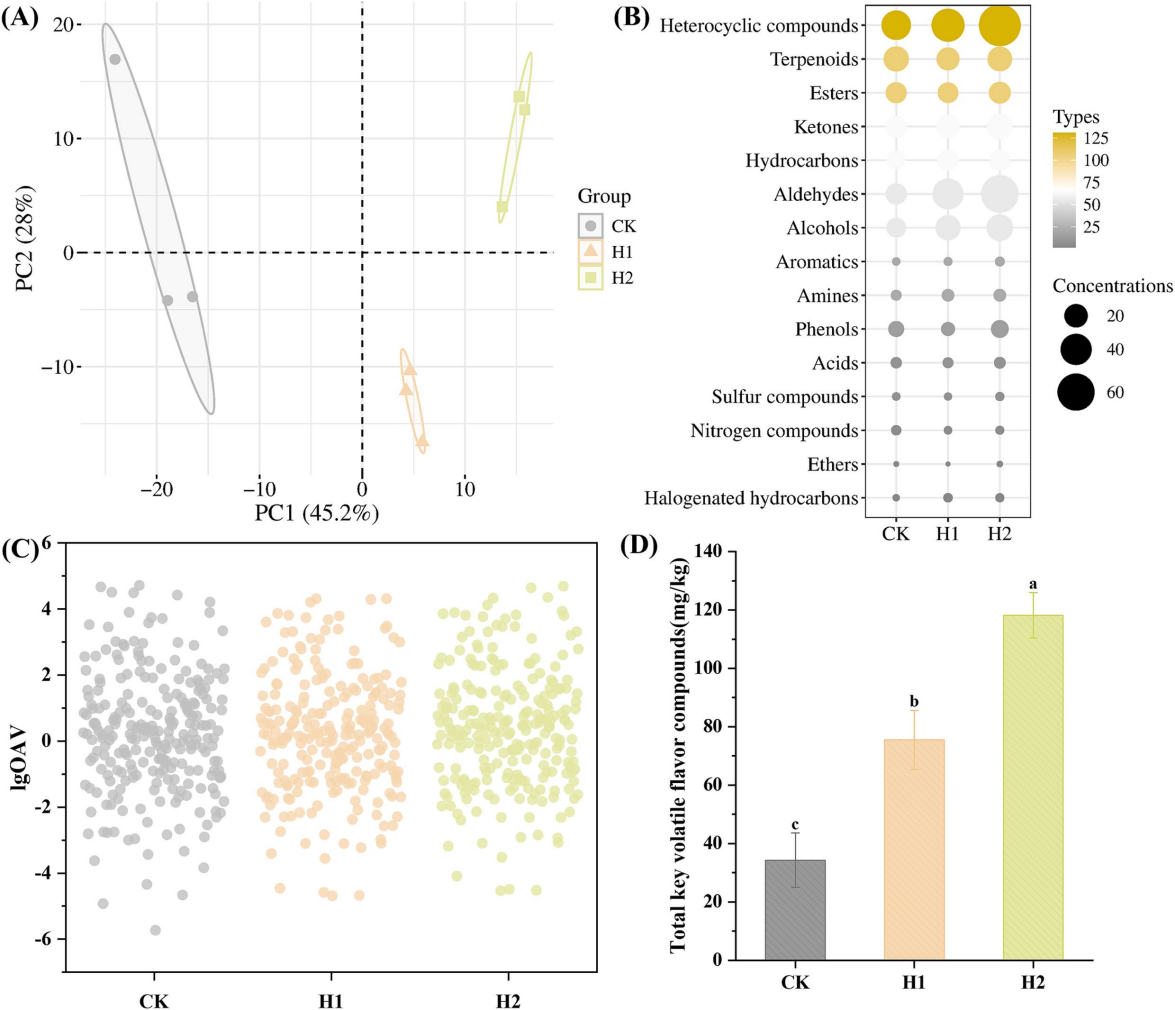
Effect of sequential heating on volatile flavor compounds in enzymatic oyster juice. **(A)** Similarity analysis of volatile flavor compounds in different treatment groups. **(B)** Total concentration in different categories. **(C)** OAV distribution of volatile flavor compounds. **(D)** Total concentration of key volatile flavor compounds (OAV ≥ 1).

Volatile flavor compounds are the crucial determinants of the flavor in aquatic products (17). In this study, the volatile flavor compounds in the enzymatic oyster juice at different treatment stage were systematically investigated. A total of 667 volatile flavor compounds were detected by HS-SPME-GC–MS in three groups, containing 131 heterocyclic compounds, 107 terpenoids, 106 esters, 64 ketones, 64 hydrocarbons, 55 aldehydes, 55 alcohols, 21 aromatics, 22 amines, 15 phenols, 9 acids, 8 sulfur compounds, 5 nitrogen compounds, 3 ethers, and 2 halogenated hydrocarbons (Supplementary Table S2). The PCA score plot about the volatile flavor compounds showed a clear separation among the CK, H1, and H2 groups (Figure 2A). The overall variation of PCA reached 71.9% with PC1 and PC2 explaining 44.2 and 27.7% of the total variance, respectively, and could well reveal the similarity of volatile flavor compounds among different groups. Similar to free amino acids, the volatile flavor compounds in the H1 and H2 groups were more different from those in the CK group, suggesting that heating had a significant impact on the concentration of volatile flavor compounds. As shown in the Figure 2B, heterocyclic compounds, aldehydes, and alcohols had the highest concentrations in all groups. Most categories of flavor volatile compounds significantly increased after heating, especially in the H2 group.

**Figure 3:**
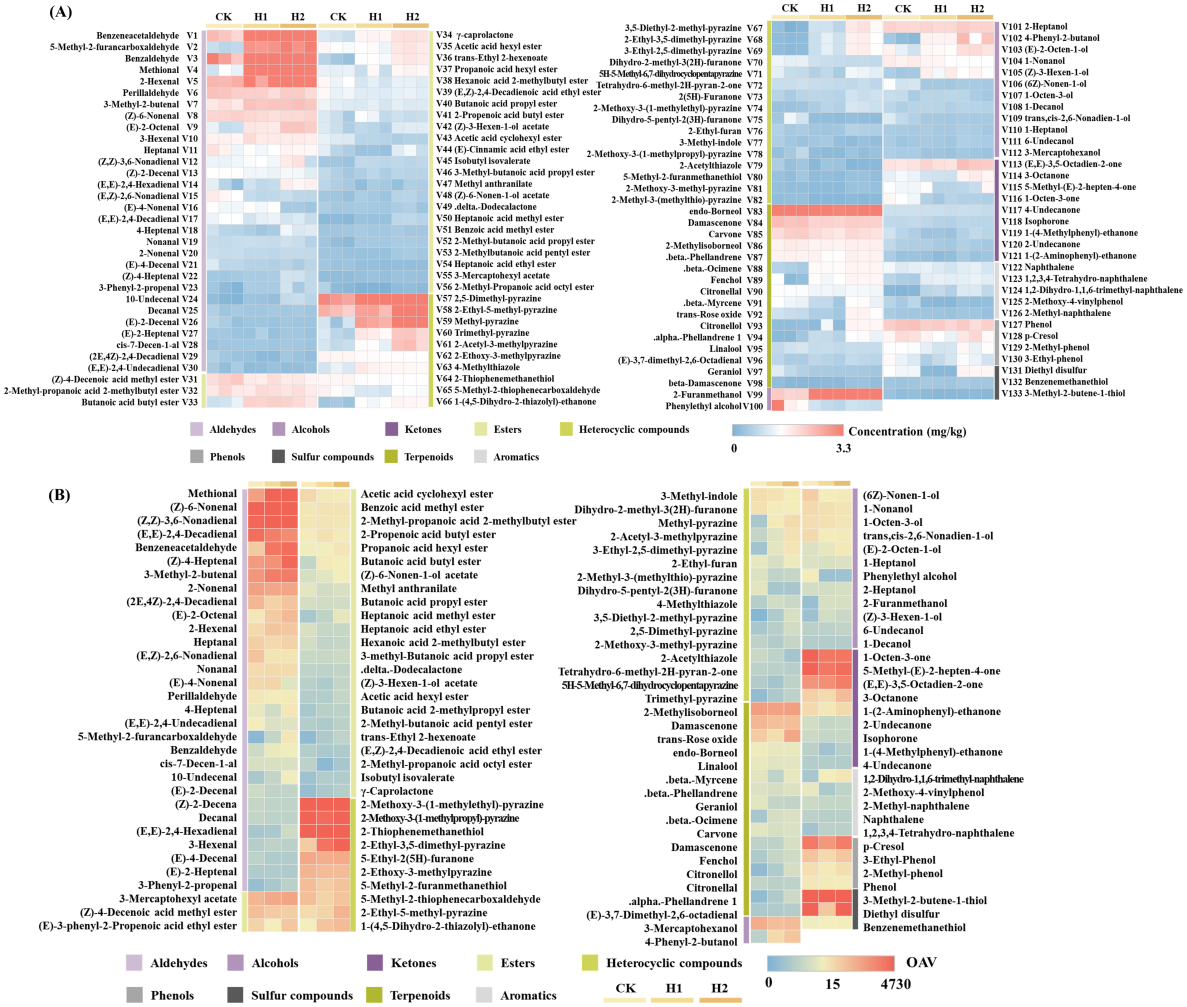
Effect of sequential heating on key volatile flavor compounds (OAV ≥ 1) in enzymatic oyster juice. **(A)** Concentration heatmap and **(B)** OAV heatmap of key volatile flavor compounds in different treatment groups.

The OAV of volatile flavor compounds can evaluate their contribution to the overall aroma of aquatic product, and the volatile flavor compounds with OAV > 1 are considered as the key flavor compounds in the aroma system (18). According to OAV ≥ 1, 133 compounds were further identified as key volatile flavor compounds, among which aldehydes, alcohols, ketones, esters, heterocyclic compounds, and terpenoids were the predominant categories (Figure 2C; Supplementary Table S2). Compared with the CK group, both heating-treated groups exhibited a denser distribution of compounds with higher OAV values, indicating an overall enhancement in odor activity after heating. Similarly, the total concentration of 133 key volatile flavor compounds gradually increased and reached their maximum value after secondary heating (Figure 2D). Similarly, the OAV of most key volatile flavor compounds peaked at the stage of secondary heating (Figure 3).

Aldehydes which are usually derived from oxidation and decomposition of fatty acids constitute one of the most important classes of key volatile flavor compounds in aquatic products (19). In this work, 30 aldehydes with OAV ≥ 1 were identified as the key volatile flavor aldehydes, most of which were significantly elevated after sequential heating. The total concentration of key aldehydes increased sharply from 9.45 mg/kg in the CK group to 56.30 mg/kg in the H2 group. Among these aldehydes, benzeneacetaldehyde, 5-methyl-2-furancarboxaldehyde, benzaldehyde, methional, and (E)-2-hexenal had the highest concentration with the maximum of 18.33, 17.95, 6.31, 4.44, and 3.28 mg/kg in the H2 group, respectively. The OAV of benzeneacetaldehyde reached 2909.38 in the H2 group, providing floral and fruity odor to oyster juice. This increase is mainly attributed to the Strecker degradation of phenylalanine under thermal conditions (20). Similarly, the OAV of methional reached its maximum value of 22206.83 after the second heating, contributing strongly roasted and vegetable odor to oyster juice. Thermal processing enhances Maillard reactions and promotes the formation of *α*-dicarbonyl compounds, thereby accelerating the conversion of methionine into methional (21). (Z,Z)-3,6-nonadienal, (Z)-4-heptenal, and 3-methyl-2-butenal also possessed their highest OAV in the H2 group, reaching 7094.4, 3234.9, and 2086.14, respectively. In addition, the OAV of some unsaturated aldehydes such as (E,Z)-2,6-nonadienal, (E,E)-2,4-decadienal, and (Z)-6-nonenal obviously decreased after heating, which could be attributed to their poor thermal stability. These polyunsaturated aldehydes are prone to further oxidation, degradation, and reactions with amino compounds under heating conditions (22).

Alcohols are another important group of volatile flavor compounds in aquatic products, and mainly derive from oxidation of lipids and degradation of amino acids (23). In this study, 14 key alcohols were identified according to OAV ≥ 1, and the total concentration of key alcohols increased sharply from 6.14 mg/kg in the CK group to 19.09 mg/kg in the H2 group. Among the key alcohols, 2-furanmethanol, phenylethyl alcohol, 2-heptanol, 4-phenyl-2-butanol, 2-octen-1-ol, (E)-1-nonanol, and (Z)-3-hexen-1-ol had the highest concentration. Although 3-mercaptohexanol had low concentration in the enzymatic oyster juice, it had the highest OAV among alcohols because of its low threshold. However, this alcohol significantly decreased after heating, probably resulting from its oxidation metabolism under high temperature. Similarly, the OAV of (6Z)-nonen-1-ol, 1-nonanol, 1-octen-3-ol, trans-cis-2,6-nonadien-1-ol, and phenylethyl alcohol also decreased after heating. After heating, 4-phenyl-2-butanol showed significant increase in OAV from 2.62 (CK group) to 198.99 (H2 group), providing strong floral, and sweet aroma to enzymatic oyster juice. This alcohol may come from Strecker degradation and Maillard reaction of phenylalanine to yield aromatic aldehydes and *α*-dicarbonyl intermediates and further to produce this compound (24). Many alcohols such as (E)-2-octen-1-ol, 2-furanmethanol, and (Z)-3-hexen-1-ol also significantly increased after heating, contributing to the flavor improvement of enzymatic oyster juice.

Ketones are mainly derived from auto-oxidation of lipids, *β*-oxidation and decarboxylation of fatty acids (25). A total of 9 ketones were selected as the key ketones (OAV ≥ 1), and their total concentration rose moderately from 1.38 mg/kg in the CK group to 1.78 mg/kg in the H2 group. (E,E)-3,5-octadien-2-one was the most abundant ketone, reaching the maximum of 1.00 mg/kg in the H2 group, and its OAV always maintained a high level, providing strong fruity, green, and grassy aroma. This ketone has also been found as the key flavor compound in the fish sauce (26). In aquatic products, 1-octen-3-one is considered as an important volatile flavor compound with fishy smell (27). In this work, 1-octen-3-one possessed the highest OAV among ketones in the CK group and was obviously reduced after heating, leading to the decrease of fishy smell in the enzymatic oyster juice. In addition, the OAV of many ketones such as 5-methyl-(E)-2-hepten-4-one, 3-octanone, 1-(2-aminophenyl)-ethanone, 2-undecanone, isophorone, and 1-(4-methylphenyl)-ethanone showed a trend of first decrease and then increase, suggesting that sequential heating could reduce the decline of substances caused by high-temperature decomposition.

Esters in the aquatic products after heating treatment are derived from the esterification reaction between acids and alcohols under high temperature (23). A total of 26 key esters (OAV ≥ 1) were identified in the enzymatic oyster juice, and showed a clear accumulation trend after sequential heating. Their total concentration increased from 3.19 mg/kg in the CK group to 3.66 mg/kg in the H1 group and 4.55 mg/kg in the H2 group. Among the esters, 4-decenoic acid methyl ester, 2-methyl-propanoic acid 2-methylbutyl ester, butanoic acid butyl ester, 5-ethyldihydro-2(3H)-furanone, acetic acid hexyl ester, and trans-ethyl 2-hexenoate had the highest concentrations. Because of the low threshold, 3-mercaptohexyl acetate had the highest OAV among all esters, and reaching the maximum of 684.28 in the H2 group, contributing to the improvement of pleasant fruity aroma in the enzymatic oyster juice. Similarly, the OAV of benzoic acid methyl ester, propanoic acid hexyl ester, butanoic acid butyl ester, (Z)-6-nonen-1-ol acetate, heptanoic acid methyl ester, and acetic acid hexyl ester obviously increased after heating, which endowed the oyster juice pleasant floral aroma. Their increase after sequential heating might result from the strong esterification of organic acids and alcohols under high temperature. Similar result has also been found in the fish after heating treatment (9). In addition, the OAV of (Z)-4-decenoic acid methyl ester, acetic acid cyclohexyl ester, 2-methyl-propanoic acid 2-methylbutyl ester, butanoic acid propyl ester, and heptanoic acid ethyl ester decreased after heating.

Heterocyclic compounds, including pyrazines, furanones, thiazoles and thiophenes, played a major role in the formation of roasted and nutty aroma in the aquatic products (28). A total of 26 key heterocyclic compounds (OAV ≥ 1) were found, and their total concentration increased markedly from 5.15 mg/kg in the CK group to 9.99 mg/kg in the H1 group and 27.04 mg/kg in the H2 group. Among the heterocyclic compounds, 2,5-dimethyl-pyrazine, and 2(5H)-furanone were the most abundant. Because of the low threshold, 2-methoxy-3-(1-methylethyl)-pyrazine had an extremely high OAV, especially reaching 48430.91 in the H2 group, providing strong almond and nutty aroma. The OAV of 2-ethyl-3,5-dimethyl-pyrazine, 2-ethyl-5-methyl-pyrazine, 1-(4,5-dihydro-2-thiazolyl)-ethanone, methyl-pyrazine, 2-acetyl-3-methylpyrazine, and 3-ethyl-2,5-dimethyl-pyrazine also obviously increased after heating and mainly provided more roasted, almond, and nutty aroma to enzymatic oyster juice. These heterocyclic compounds are typical Maillard products formed from reactions between amino acids and reducing sugars, as well as from thiamine and sulfur-containing precursors under thermal conditions (29).

Terpenoids were another major category of substances that constitute the characteristic aroma of enzymatic oyster juice. There were 16 key terpenoids (OAV ≥ 1) and their total concentration changed slightly, compared with other volatile flavor compounds. Among the terpenoids, endo-borneol, 2,6,6-trimethyl-1,3-cyclohexadiene-1-carboxaldehyde, and carvone had the highest concentration and all showed the trend of first decrease and then increase. As one terpenoid with the highest OAV, 2-methylisoborneol significantly decreased after heating, contributing to the decrease of unpleasant earthy and musty smell. The OAV of trans-rose oxide with floral significantly increased after secondary heating, reaching 609.50 at H2 group. Similarly, the OAV of beta-ocimene, (Z)-3,7-dimethyl-1,3,6-octatriene, (E)-1-(2,6,6-trimethyl-1,3-cyclohexadien-1-yl)-2-buten-1-one, fenchol, citronellol, and alpha-phellandrene 1 obviously increased after heating, endowing stronger floral, herbal, and sweet aroma to enzymatic oyster juice. These terpenoids probably originate from endogenous terpenes and carotenoid derivatives in oysters as well as the release of glycosidically bound precursors during heating (30).

Aromatics are generally minor in concentration but exhibit a strong increase in odor activity after heating, mainly providing aromatic, balsamic and tarry odors (7, 31). A total of 5 key aromatics (OAV ≥ 1) were found, and their total concentration increased from 0.26 mg/kg in the CK group to 0.34 mg/kg in the H1 group and 0.40 mg/kg in the H2 group. The OAV of 1,2-dihydro-1,1,6-trimethyl-naphthalene also gradually increased after heating, providing sweet and aromatic aroma. These aromatics are mainly produced through high temperature cyclization and dehydrogenation of unsaturated fatty acids and terpenoids, as well as through secondary reactions of phenolic and Maillard-derived intermediates (32). Besides, some aromatics with unpleasant odor, such as 2-methoxy-4-vinylphenol and naphthalene significantly reduced after heating, contributing to the flavor improvement of enzymatic oyster juice.

Phenols are primarily responsible for phenolic, smoky, medicinal and slightly animalic odors (33). A total of 4 key phenols (OAV ≥ 1) were identified. Different from other volatile flavor compounds terpenoids, their total concentration significantly decreased after heating. Phenol and 3-ethyl-phenol possessing unpleasant odor including medicinal phenol, and musty smell significantly reduced after heating, helping to improve the flavor of enzymatic oyster juice.

Sulfur compounds are the main component that endow characteristic flavor to aquatic products (34). In this study, there were 3 key sulfur compounds (OAV ≥ 1) in the enzymatic oyster juice, and their total concentration increased from 0.22 mg/kg in the CK group to 0.34 mg/kg in the H2 group. Although these sulfur compounds had low concentrations, but exhibited extremely high OAV which were crucial contributors to the whole flavor of enzymatic oyster juice. The 3-methyl-2-butene-1-thiol, diethyl disulfur, and benzenemethanethiol mainly possessing onion, garlic, and sulfur smell were the characteristic flavor compounds for oyster juice. These sulfur compounds mainly derive from thermal degradation and Strecker degradation of sulfur-containing amino acids such as cysteine methionine, glutathione, and thiamine, followed by oxidation, condensation and rearrangement of intermediate thiols to di- and trisulfides (35).

**Figure 4:**
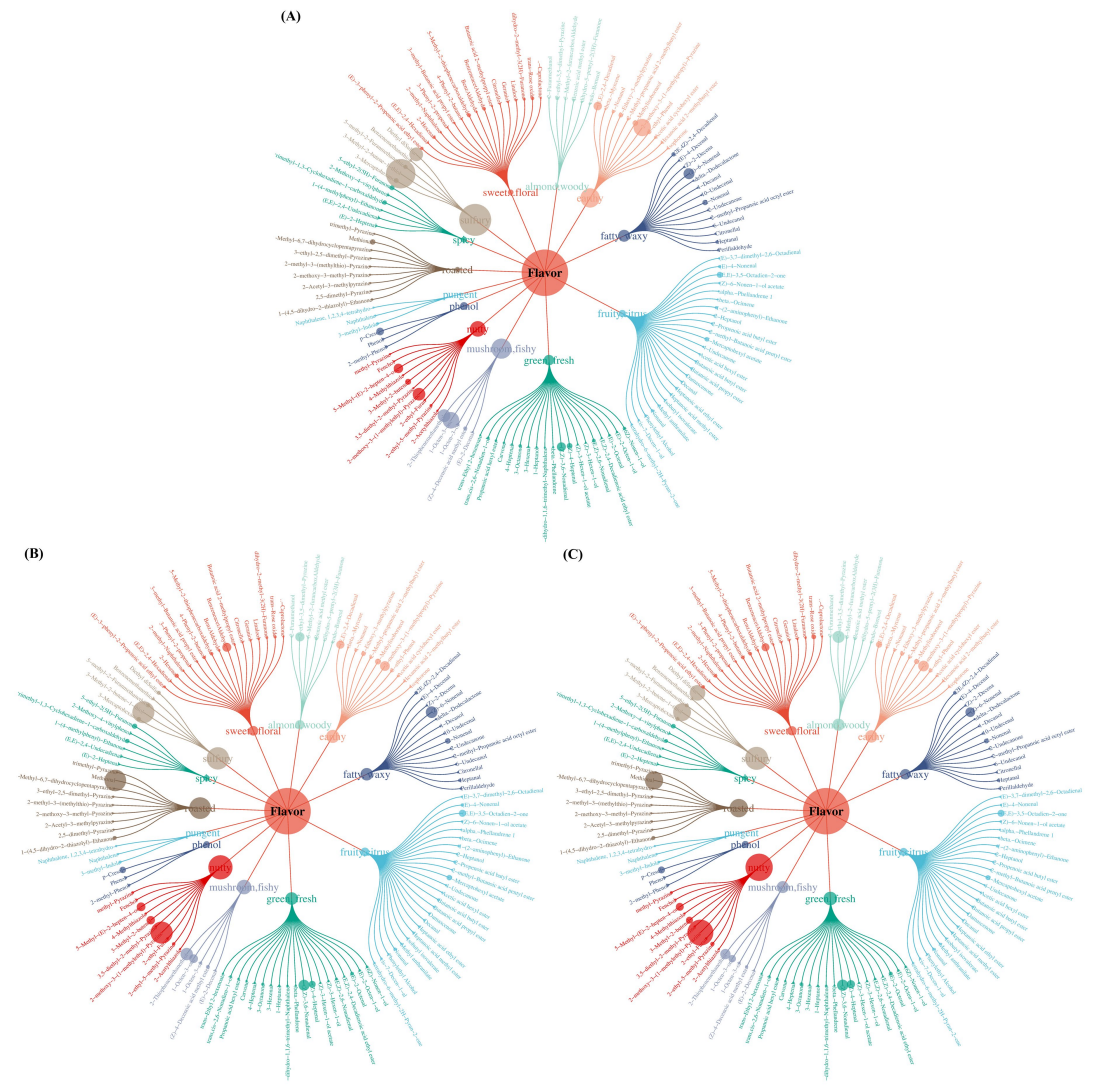
Odor profile of enzymatic oyster juice in the **(A)** CK, **(B)** H1, and **(C)** H2 groups.

In order to further clarify the odor composition of the oyster juice at different stage, the key volatile flavor compounds with OAV ≥ 1 were classified according to their odor descriptions, and the circle networks were draw (Figure 4). The odor profile of oyster juice in the whole process mainly included green, fruity, sweet, fatty, earthy, almond, nutty, roasted, sulfury, and mushroom odor. After heating, the OAV of most volatile compounds with pleasant flavor significantly increased. Notably, the OAV of volatile flavor compounds with nutty odor sharply increased from 12600.81 in the CK group to 53926.05 in the H2 group. Similarly, the characteristic flavors such as roasted and almond odor gradually increased after heating. The OAV of volatile flavor compounds with roasted and almond odor obviously changed from 823.86 and 220.31 in the CK group to 22629.51 and 8542.57 in the H2 group, respectively. Moreover, other pleasant flavor such as fruity, green, and sweet odor also enhanced after heating. On the contrary, the unpleasant flavor such as fishy, earthy, fatty, sulfury, and spicy odor was obviously weakened after heating. The OAV of volatile flavor compounds with mushroom and fishy odor significantly decreased from 22595.43 in the CK group to 8374.24 in the H2 group, indicating heating could effectively reduce the fishy-like smell. The OAV of earthy and sulfury volatile flavor compounds also decreased from 20462.13 and 61623.18 in the CK group to 9131.35 and 37333.92 in the H2 group, respectively. In general, sequential heating enhanced the pleasant odor and weakened the unpleasant odor, consequently improving the whole flavor of enzymatic oyster juice.

### 3.3 Correlation analysis of key volatile flavor compounds with free amino acids during processing of oyster juice

**Figure 5:**
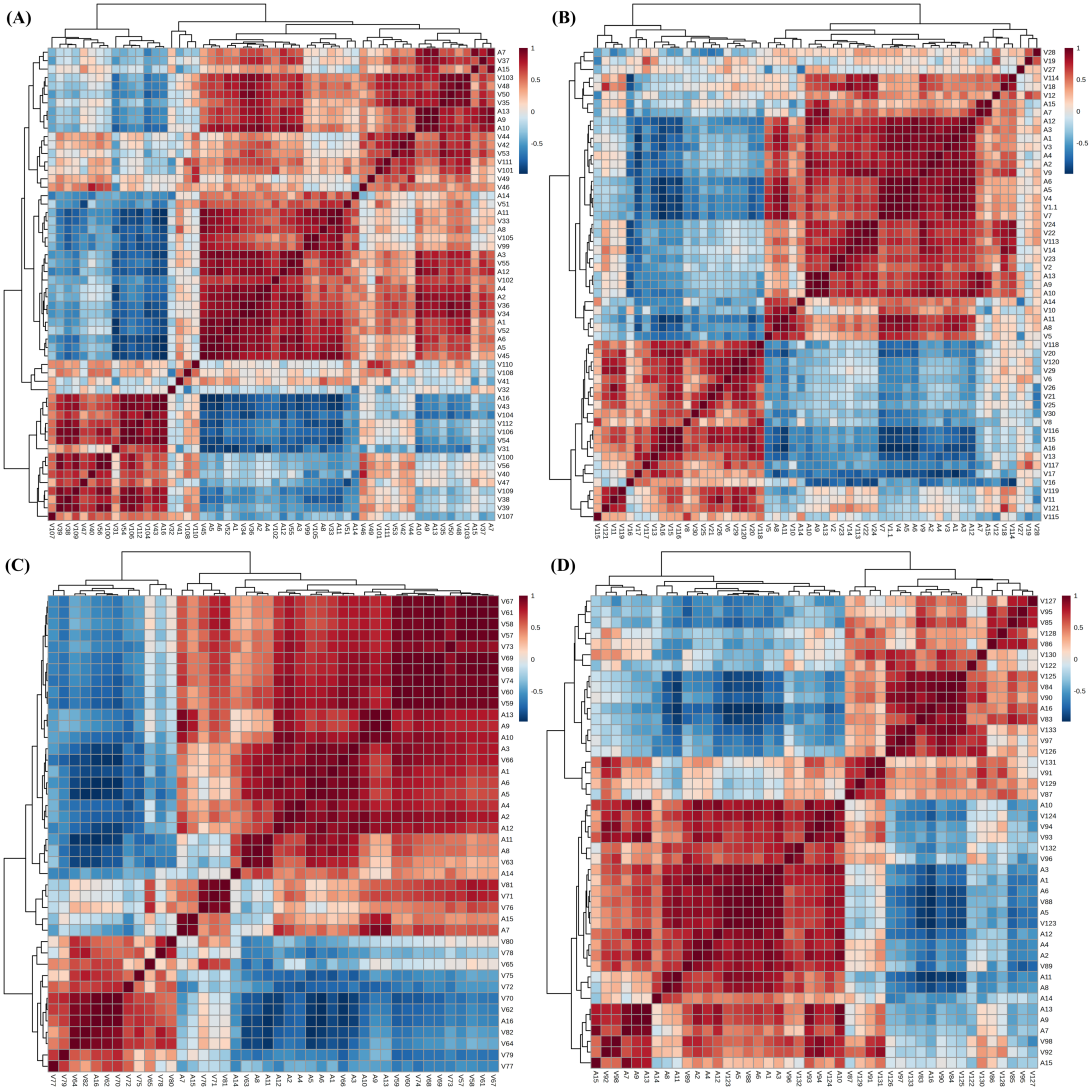
Correlation heatmap of between free amino acids with **(A)** alcohols and esters, **(B)** aldehydes and ketones, **(C)** heterocyclic compound, and **(D)** other key volatile flavor compounds.

In order to further clarify the effect mechanism of sequential heating on the flavor improvement of enzymatic oyster juice, the correlation analysis of volatile flavor compounds and free amino acids was performed (Figure 5). Previous studies have shown that Phe can undergo the Strecker degradation reaction under heating conditions, resulting in the formation of benzeneacetaldehyde (36). The degradation process of Phe may also lead to the production of benzaldehyde, and Tyr can also generate aromatic Strecker-type aldehydes (37, 38). In this study, benzeneacetaldehyde and benzaldehyde were, respectively, positively correlated with Tyr and Phe, suggesting that aromatic amino acids may contribute to the formation of aromatic aldehydes through Strecker degradation or related thermal reactions. Methional and 4-methylthiazole were significantly positively correlated with Met, indicating that sulfur-containing amino acids might serve as important precursors for sulfur compounds. In addition, Ser, Thr, Leu, Ile, Val, and Lys were positively correlated with several pyrazines. In particular, Leu was strongly correlated with 2-methoxy-3-(1-methylethyl)-pyrazine, 2-ethyl-3,5-dimethylpyrazine, and 3-ethyl-2,5-dimethylpyrazine, suggesting that branched-chain amino acids may be involved in the formation of alkyl-substituted pyrazines. The positive correlations of Lys, Ser, and Thr with methylpyrazine and trimethylpyrazine further supported the important role of the Maillard reaction in improving the flavor quality of oyster juice (39, 40). Furthermore, some volatile compounds produced by lipid oxidation (22), such as (E)-2-octenal, 3-hexenal, (E,E)-2,4-hexadienal, and 2-hexenal, were positively correlated with some free amino acids such as Asp., Gly, Ala, and Met. These results indicated that heating could simultaneously promote protein degradation and lipid oxidation. Sequential heating promoted protein degradation, amino acid release, Maillard reaction, Strecker degradation and lipid oxidation, and these reactions collectively contributed to the improvement of flavor and aroma in oyster juice.

### 3.4 Construction of differential formation pathways of key flavor compounds during processing of oyster juice

**Figure 6:**
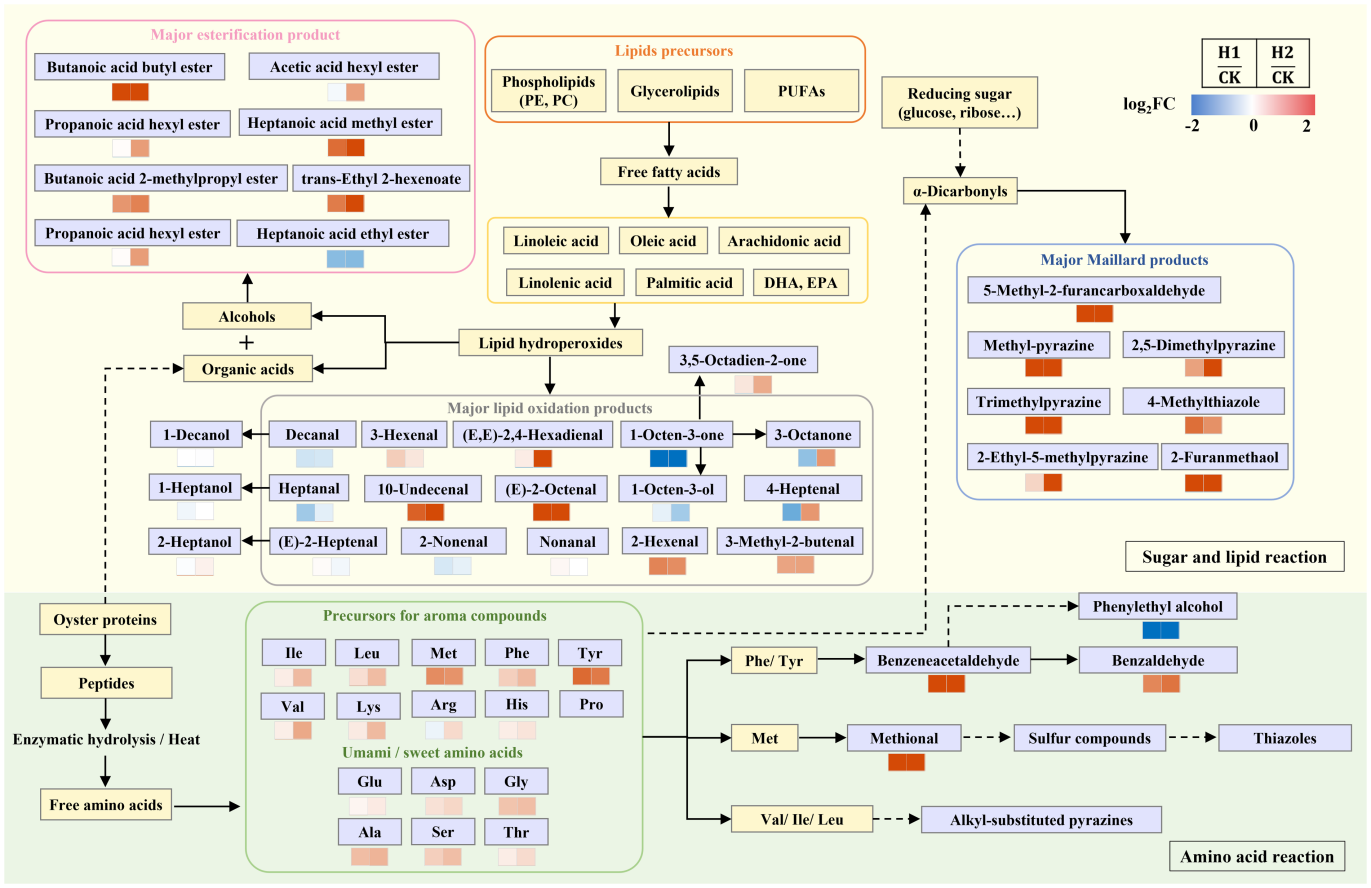
Formation pathways of key volatile flavor compounds and free amino acids during the processing of oyster.

In order to better describe the improvement mechanism of key volatile flavor compounds and free amino acids of enzymatic oyster juice after sequential heating, the differential formation pathways of key flavor compounds were constructed (Figure 6). Oysters are rich in lipids, unsaturated fatty acids and proteins, which provides a solid foundation for the flavor formation of oyster juice (41). The enzymatic breakdown and heating processes promoted the production of unsaturated fatty acids, mainly including linoleic acid, oleic acid, arachidonic acid, linolenic acid, palmitic acid, DHA, and EPA (42). In particular, heating caused these unsaturated fatty acids to convert into lipid hydroperoxides (22), which further broke down to produce small molecular flavor compounds such as 3-hexenal, (E,E)-2,4-hexadienal, (E)-2-octenal, 10-undecenal, 4-heptenal, 2-hexenal, 3-methyl-2-butenal, 3-octanone, 1-octen-3-ol, and 1-octen-3-one. These small molecular volatile compounds generally increased significantly after heating, especially the second stage of heating. Moreover, lipids could also undergo oxidation and decomposition to produce organic acids (43), which could undergo esterification reactions with alcohols under heating conditions to form esters mainly including butanoic acid butyl ester, acetic acid hexyl ester, propanoic acid hexyl ester, heptanoic acid methyl ester, butanoic acid 2-methylpropyl ester, trans-ethyl 2-hexenoate, and propanoic acid hexyl ester. Additionally, these organic acids might also originate from the breakdown of proteins or peptides. Similarly, free amino acids are mainly produced through the breakdown of proteins and peptides. Amino acids usually serve as taste components in aquatic products (44). After heating, almost all amino acids increased significantly, especially after the second heating, contributing to the important sweet and umami taste to oyster juice. Amino acids could also undergo the Maillard reaction with reducing sugars (10), resulting in the production of volatile compounds such as 5-methyl-2-furancarboxaldehyde, methyl-pyrazine, 2,5-dimethylpyrazine, trimethylpyrazine, and 2-ethyl-5-methylpyrazine. These Maillard products significantly increased after the second heating, enhancing the meaty flavor and roasted aroma of the oyster juice. Under heating condition, Phe or Tyr could produce benzeneacetaldehyde, which could also be further transformed into benzaldehyde and phenylethyl alcohol (37, 38). Met is also an important precursor amino acid for the formation of volatile compounds (45). Met could generate Methional, which in turn could form sulfides and thiazoles. Val, Ile and Leu can produce alkyl-substituted pyrazines, which play a significant role in enhancing the flavor of oyster juice (46). In this study, heating promoted the oxidation and decomposition of lipids and proteins, thereby contributing to the improvement of volatile compounds and free amino acids.

### 3.5 Flavor improvement mechanism of enzymatic oyster juice by sequential heating

**Figure 7:**
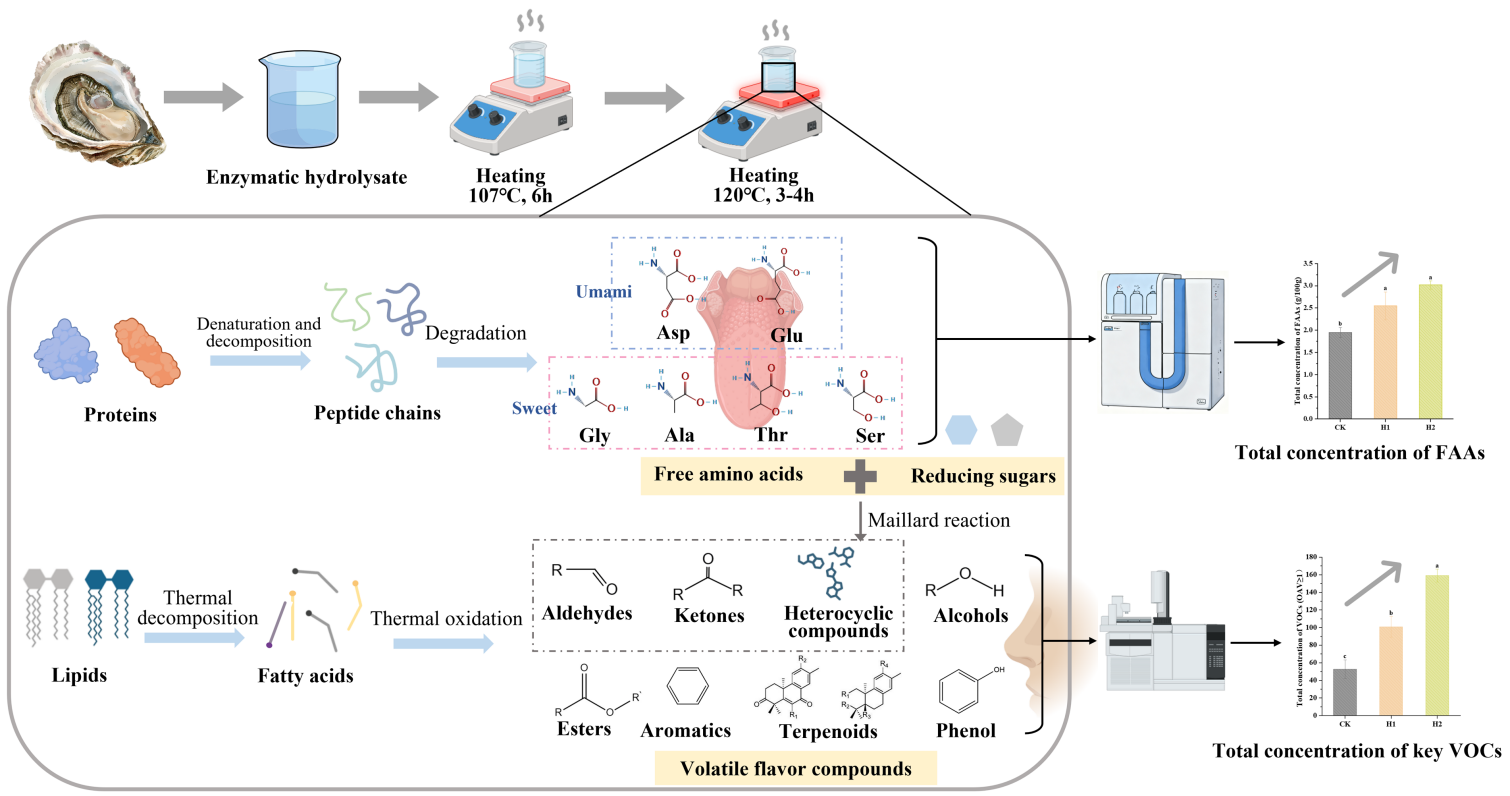
Flavor improvement mechanism of enzymatic oyster juice by sequential heating.

The flavor improvement mechanisms of enzymatic oyster juice by the sequential heating strategy were further summarized (Figure 7). Although protease hydrolysis can produce more free amino acids or peptides in the oyster juice, it is unable to release abundant volatile aroma compounds, compared with that by traditional processing method. In this study, sequential heating could significantly improve the flavor of enzymatic oyster juice. As the main taste compounds, the free amino acids were markedly elevated after sequential heating, probably because high temperature could easily break down the proteins or peptides. In particularly, it significantly increased the concentrations of free amino acids that contribute to the umami and sweet flavors, such as Asp., Glu, Thr, Gly, and Ala. Free amino acids could also serve as important precursors for the change in volatile flavor compounds. They can participate in the Maillard reaction with reducing sugars to generate aldehydes, ketones and other compounds with pleasant odors and also to reduce the unpleasant volatile flavor compounds (9). Furthermore, the decomposition of lipids under heating conditions was also one of the important ways for production of volatile flavor compounds, especially the oxidation decomposition of fatty acids.

Based on the results of this study, the contents of amino acid nitrogen, total free amino acids, and volatile flavor compounds in the enzymatic oyster juice after sequential heating were significantly higher than those in the oyster juice after a single heating. Importantly, the second stage of heating provides a more suitable temperature for the Maillard reaction to occur, thereby generating more substances with meaty and nutty aromas, enhancing the rich aroma of the oyster juice and improving its quality (15, 47). Therefore, sequential heating can be considered as a practical and targeted approach to optimize the taste and odor profile, and is finally used to improve the sensory quality of oyster juice.

## 4 Conclusion

Heating treatment resulted in a significant increase in the concentration of free amino acids which reached the highest level after the secondary heating. The production of free amino acids with sweet and umami taste were significantly promoted by sequential heating, improving the taste of enzymatic oyster juice. A total of 133 key volatile flavor compounds with OAV ≥ 1 were screened out, while aldehydes, alcohols, ketones, esters, heterocyclic compounds, and terpenoids provided the main aroma of enzymatic oyster juice. According to odor profile of enzymatic oyster juice, the pleasant volatile flavor such as nutty, almond, fruity, green, and sweet odor was significantly enhanced by sequential heating, while the unpleasant volatile flavor such as fishy, earthy, fatty, sulfury, and spicy odor was significantly reduced. The taste and odor of enzymatic oyster juice were improved by sequential heating mainly through thermal reaction including Maillard reaction, Strecker degradation, as well as oxidation and degradation of lipids and proteins. Sequential heating is an effective method to improve the content of free amino acids and volatile flavor compounds in the enzymatic oyster juice, and further regulate its flavor.

## Data Availability

The original contributions presented in the study are included in the article/Supplementary material, further inquiries can be directed to the corresponding authors.
