# Supplementary material for: Flavor improvement of enzymatic oyster juice by sequential heating through the accumulation of free amino acids and volatile flavor compounds
Source: Front Nutr. 2026 Jul 17;13:1907081. doi: 10.3389/fnut.2026.1907081 (PMC13423985; doi:10.3389/fnut.2026.1907081)
Supplement: Supplementary file 1 [file Data_Sheet_1.docx]

Supplementary Material

**Table S1** Change in the concentrations of free amino acids (g/100g) in enzymatic oyster juice after sequential heating.

| Free amino acid | CK | H1 | H2 |
| --- | --- | --- | --- |
| Asp | 0.13±0.01^b^ | 0.16±0.01^a^ | 0.17±0.01^a^ |
| Thr | 0.15±0.01^b^ | 0.17±0.01^ab^ | 0.19±0.01^a^ |
| Ser | 0.11±0.01^b^ | 0.16±0.02^a^ | 0.19±0.01^a^ |
| Glu | 0.21±0.01^b^ | 0.23±0.01^ab^ | 0.25±0.01^a^ |
| Gly | 0.12±0.02^b^ | 0.2±0.01^a^ | 0.2±0.01^a^ |
| Ala | 0.17±0.01^b^ | 0.28±0.02^a^ | 0.3±0.01^a^ |
| Val | 0.12±0.01^a^ | 0.14±0.12^a^ | 0.23±0.01^a^ |
| Met | 0.06±0.01^b^ | 0.14±0.04^a^ | 0.13±0.01^a^ |
| Ile | 0.11±0.01^b^ | 0.13±0.04^ab^ | 0.19±0.01^a^ |
| Leu | 0.16±0.02^b^ | 0.21±0.04^ab^ | 0.27±0.01^a^ |
| Tyr | 0.07±0.01^b^ | 0.21±0.03^a^ | 0.19±0.01^a^ |
| Phe | 0.11±0.01^b^ | 0.16±0.02^a^ | 0.19±0.01^a^ |
| Lys | 0.17±0.01^b^ | 0.2±0.07^ab^ | 0.28±0.01^a^ |
| His | 0.04±0.01^a^ | 0.05±0.01^a^ | 0.05±0.01^a^ |
| Arg | 0.14±0.02^a^ | 0.13±0.08^a^ | 0.19±0.01^a^ |
| Pro | 0.08±0.01 | - | - |
| Total content | 1.95±0.12^b^ | 2.55±0.32^a^ | 3.02±0.09^a^ |

Data in the same row labelled with different letters are statistically different at *p* < 0.05

**Table S2** Change in the OAV of key volatile flavor compounds (OAV ≥ 1) in enzymatic oyster juice after sequential heating.

| No. | Compounds | RI | Odor | Threshold  (mg/kg) | CK | H1 | H2 |  |
| --- | --- | --- | --- | --- | --- | --- | --- | --- |
| Aldehydes | | | | | | | | |
| 1 | Methional | 906 | roasted, vegetable, creamy | 0.0002 | 782.33 | 19331.96 | 22206.83 |  |
| 2 | (Z)-6-Nonenal | 1103 | waxy, green, fresh | 0.00014 | 5471.07 | 4730.19 | 4959.43 |  |
| 3 | (Z,Z)-3,6-Nonadienal | 1100 | green, fatty, fresh | 0.00005 | 3315.97 | 3992.89 | 7094.39 |  |
| 4 | (E,E)-2,4-Decadienal | 1320 | dusty, waxy, fatty | 0.0005 | 2810.97 | 1915.00 | 1397.71 |  |
| 5 | Benzeneacetaldehyde | 1045 | floral, fruity, honey | 0.0063 | 155.34 | 2422.52 | 2909.38 |  |
| 6 | (Z)-4-Heptenal | 900 | green, fatty, creamy | 0.000025 | 907.01 | 1188.01 | 3234.96 |  |
| 7 | 3-Methyl-2-butenal | 782 | nutty, almond, sweet | 0.0005 | 1017.04 | 2047.43 | 2086.14 |  |
| 8 | 2-Nonenal | 1161 | fatty, green, waxy | 0.0001 | 748.48 | 601.12 | 654.07 |  |
| 9 | (2E,4Z)-2,4-Decadienal | 1295 | fatty, green, waxy | 0.00007 | 355.74 | 115.25 | 215.36 |  |
| 10 | (E)-2-Octenal | 1057 | green, fresh, herbal | 0.003 | 34.48 | 157.44 | 327.64 |  |
| 11 | 2-Hexenal | 853 | sweet, almond, fruity, fruity, green, vegetable | 0.017 | 80.27 | 206.48 | 193.17 |  |
| 12 | Heptanal | 901 | fatty, green, fresh | 0.0028 | 129.50 | 77.35 | 111.58 |  |
| 13 | (E,Z)-2,6-Nonadienal | 1155 | green, fresh | 0.0015 | 212.85 | 42.44 | 42.61 |  |
| 14 | Nonanal | 1100 | citrus, orange peel | 0.001 | 70.22 | 74.99 | 70.36 |  |
| 15 | (E)-4-Nonenal | 1104 | fruity | 0.0022 | 85.39 | 98.44 | 11.19 |  |
| 16 | Perillaldehyde | 1274 | fatty, fresh, grassy | 0.03 | 33.50 | 25.61 | 27.56 |  |
| 17 | 4-Heptenal | 899 | green, fatty | 0.0042 | 14.80 | 6.65 | 32.86 |  |
| 18 | (E,E)-2,4-Undecadienal | 1430 | spicy, fatty, buttery | 0.001 | 22.16 | 9.62 | 11.07 |  |
| 19 | 5-Methyl-2-furancarboxAldehyde | 964 | almond, caramel, buttery | 0.5 | 0.13 | 4.18 | 35.89 |  |
| 20 | Benzaldehyde | 962 | sweet, almond, fruity | 0.35 | 6.22 | 15.69 | 18.04 |  |
| 21 | cis-7-Decen-1-al | 1212 | citrus, green, fresh | 0.0022 | 8.55 | 9.08 | 9.33 |  |
| 22 | 10-Undecenal | 1297 | waxy, fatty, citrus | 0.0035 | 1.21 | 4.05 | 18.54 |  |
| 23 | (E)-2-Decenal | 1263 | mushroom, waxy, earthy | 0.005 | 6.17 | 3.33 | 3.65 |  |
| 24 | (Z)-2-Decenal | 1252 | fatty, waxy | 0.05 | 4.48 | 3.40 | 3.33 |  |
| 25 | Decanal | 1206 | citrus, sweet, floral | 0.007 | 3.98 | 3.07 | 3.16 |  |
| 26 | (E,E)-2,4-Hexadienal | 910 | sweet, green, floral | 0.06 | 1.06 | 1.23 | 6.13 |  |
| 27 | 3-Hexenal | 810 | green, tomato, fruity | 0.16 | 2.15 | 3.17 | 2.62 |  |
| 28 | (E)-4-Decenal | 1198 | fatty, aldehydic, citrus | 0.025 | 3.11 | 1.68 | 1.76 |  |
| 29 | (E)-2-Heptenal | 957 | spicy, fatty | 0.013 | 1.60 | 1.66 | 1.49 |  |
| 30 | 3-Phenyl-2-propenal | 1273 | sweet, aromatic, honey | 0.024 | 0.38 | 1.21 | 2.63 |  |
| Alcohols | | | | | | | | |
| 31 | 3-Mercaptohexanol | 1127 | sulfury, fruity, tropical | 0.00006 | 512.88 | 271.96 | 313.65 |  |
| 32 | 4-Phenyl-2-butanol | 1262 | floral, sweet | 0.0043 | 2.62 | 80.24 | 198.99 |  |
| 33 | (6Z)-Nonen-1-ol | 1171 | fresh, green, waxy | 0.001 | 136.80 | 23.07 | 26.88 |  |
| 34 | 1-Nonanol | 1168 | earthy, dusty, fatty | 0.0053 | 75.17 | 54.40 | 41.29 |  |
| 35 | 1-Octen-3-ol | 980 | mushroom, fatty, fruity | 0.001 | 58.06 | 50.94 | 35.34 |  |
| 36 | trans,cis-2,6-Nonadien-1-ol | 1170 | green, fresh, fatty | 0.001 | 75.54 | 24.28 | 26.84 |  |
| 37 | (E)-2-Octen-1-ol | 1067 | green, citrus, vegetable | 0.02 | 9.77 | 13.96 | 29.95 |  |
| 38 | 1-Heptanol | 970 | green, grassy | 0.003 | 9.56 | 8.81 | 9.58 |  |
| 39 | Phenylethyl Alcohol | 1116 | fruity, floral, sweet | 0.14 | 25.46 | 0.51 | 0.58 |  |
| 40 | 2-Heptanol | 900 | fruity | 0.1 | 7.28 | 7.16 | 8.10 |  |
| 41 | 2-Furanmethanol | 856 | almond, sweet, bread | 1.9 | 0.43 | 8.54 | 8.45 |  |
| 42 | (Z)-3-Hexen-1-ol | 857 | fresh, green | 0.05 | 0.53 | 6.81 | 5.08 |  |
| 43 | 6-Undecanol | 1277 | waxy, fatty | 0.0086 | 2.82 | 2.77 | 3.73 |  |
| 44 | 1-Decanol | 1271 | fatty, waxy, floral | 0.023 | 2.01 | 2.03 | 1.99 |  |
| Ketones | | | | | | | | |
| 45 | 1-Octen-3-one | 976 | mushroom | 0.000016 | 14422.24 | 2283.82 | 2412.95 |  |
| 46 | 5-Methyl-(E)-2-hepten-4-one | 972 | nutty | 0.00005 | 3757.13 | 2270.89 | 2902.76 |  |
| 47 | (E,E)-3,5-Octadien-2-one | 1073 | fruity, green, grassy | 0.0005 | 1049.88 | 1280.00 | 2006.33 |  |
| 48 | 3-Octanone | 986 | fresh, herbal, sweet | 0.0013 | 127.08 | 69.93 | 284.24 |  |
| 49 | 1-(2-aminophenyl)-Ethanone | 1308 | fruity, sweet | 0.00027 | 75.21 | 33.53 | 75.38 |  |
| 50 | 2-Undecanone | 1294 | fatty, waxy, fruity | 0.0062 | 7.13 | 4.45 | 4.97 |  |
| 51 | Isophorone | 1123 | earthy, woody, musty | 0.011 | 5.66 | 4.28 | 4.72 |  |
| 52 | 1-(4-methylphenyl)-Ethanone | 1183 | spicy, pepper, pea | 0.021 | 2.85 | 0.77 | 2.43 |  |
| 53 | 4-Undecanone | 1208 | fruity | 0.041 | 2.06 | 1.95 | 1.83 |  |
| Esters | | | | | | | | |
| 54 | 3-Mercaptohexyl acetate | 1248 | fruity | 0.00002 | 377.14 | 592.20 | 684.28 |  |
| 55 | (Z)-4-Decenoic acid methyl ester | 1323 | fishy, fruity | 0.003 | 264.18 | 162.73 | 136.44 |  |
| 56 | (E)-3-phenyl-2-Propenoic acid ethyl ester | 1463 | floral, sweet | 0.0007 | 104.33 | 21.23 | 185.91 |  |
| 57 | Acetic acid cyclohexyl ester | 1043 | earthy, musty | 0.0016 | 81.79 | 31.26 | 24.71 |  |
| 58 | Benzoic acid methyl ester | 1097 | almond, floral | 0.00052 | 37.16 | 45.51 | 46.19 |  |
| 59 | 2-Methyl-propanoic acid 2-methylbutyl ester | 1016 | musty, fruity, tropical | 0.014 | 39.61 | 40.48 | 36.85 |  |
| 60 | 2-Propenoic acid butyl ester | 896 | fruity | 0.0029 | 25.83 | 50.58 | 27.31 |  |
| 61 | Propanoic acid hexyl ester | 1108 | green, fruity | 0.008 | 18.34 | 18.90 | 38.97 |  |
| 62 | Butanoic acid butyl ester | 996 | fruity, green | 0.028 | 3.97 | 28.71 | 23.15 |  |
| 63 | (Z)-6-Nonen-1-ol acetate | 1308 | fruity, green | 0.002 | 8.47 | 11.94 | 21.51 |  |
| 64 | Methyl anthranilate | 1348 | fruity, flowery | 0.003 | 12.87 | 6.98 | 8.92 |  |
| 65 | Butanoic acid propyl ester | 899 | fruity, sweet | 0.018 | 7.81 | 6.82 | 6.98 |  |
| 66 | Heptanoic acid methyl ester | 1024 | fruity, sweet, floral | 0.004 | 1.14 | 3.49 | 13.71 |  |
| 67 | Heptanoic acid ethyl ester | 1097 | wine, fruity | 0.002 | 8.97 | 4.73 | 4.63 |  |
| 68 | Hexanoic acid 2-methylbutyl ester | 1247 | musty, ethereal | 0.032 | 7.27 | 3.64 | 4.43 |  |
| 69 | 3-methyl-Butanoic acid propyl ester | 951 | sweet, fruity | 0.0087 | 4.86 | 4.71 | 5.56 |  |
| 70 | .delta.-Dodecalactone | 1720 | fatty, creamy, soapy | 0.0076 | 3.41 | 3.08 | 3.80 |  |
| 71 | (Z)-3-Hexen-1-ol acetate | 1006 | fresh, green, sweet | 0.031 | 2.55 | 1.54 | 4.14 |  |
| 72 | Acetic acid hexyl ester | 1013 | fruity, green | 0.115 | 2.05 | 1.94 | 4.24 |  |
| 73 | Butanoic acid 2-methylpropyl ester | 954 | sweet, fruity | 0.0094 | 1.14 | 2.56 | 2.99 |  |
| 74 | 2-methyl-Butanoic acid pentyl ester | 1142 | fruity, tropical | 0.0086 | 1.91 | 1.48 | 2.86 |  |
| 75 | trans-Ethyl 2-hexenoate | 1049 | green, fruity, vegetable | 0.14 | 0.20 | 1.52 | 3.29 |  |
| 76 | (E,Z)-2,4-Decadienoic acid ethyl ester | 1479 | green, waxy, fruity | 0.1 | 2.05 | 1.28 | 1.45 |  |
| 77 | 2-methyl-Propanoic acid octyl ester | 1344 | fatty, waxy, soapy | 0.006 | 2.90 | 0.16 | 1.06 |  |
| 78 | Isobutyl isovalerate | 1005 | fruity, sweet | 0.034 | 0.56 | 1.75 | 1.69 |  |
| 79 | γ-Caprolactone | 1226 | sweet, caramel | 0.26 | 0.37 | 1.23 | 2.14 |  |
| Heterocyclic compounds | | | | | | | |  |
| 80 | 2-methoxy-3-(1-methylethyl)-Pyrazine | 1097 | nutty, almond | 0.000002 | 7729.66 | 20352.91 | 48430.91 |  |
| 81 | 2-methoxy-3-(1-methylpropyl)-Pyrazine | 1175 | musty, spicy, pepper | 0.000002 | 15969.22 | 3018.60 | 6311.91 |  |
| 82 | 2-Thiophenemethanethiol | 1105 | fishy, roasted | 0.00004 | 7844.78 | 5440.54 | 5785.86 |  |
| 83 | 2-ethyl-3,5-dimethyl-Pyrazine | 1084 | almond, roasted, nutty | 0.00004 | 152.62 | 2580.59 | 8435.75 |  |
| 84 | 5-ethyl-2(5H)-Furanone | 966 | spicy | 0.0097 | 583.27 | 490.43 | 559.95 |  |
| 85 | 2-Ethoxy-3-methylpyrazine | 1065 | earthy, roasted, almond | 0.0008 | 402.90 | 312.62 | 303.69 |  |
| 86 | 5-methyl-2-Furanmethanethiol | 995 | sulfury, roasted, coffee | 0.00005 | 288.33 | 144.92 | 220.75 |  |
| 87 | 5-Methyl-2-thiophenecarboxaldehyde | 1118 | sweet, woody | 0.001 | 262.06 | 107.60 | 206.58 |  |
| 88 | 2-ethyl-5-methyl-Pyrazine | 1005 | nutty, grassy, roasted | 0.016 | 72.47 | 100.52 | 397.98 |  |
| 89 | 1-(4,5-dihydro-2-thiazolyl)-Ethanone | 1106 | roasted, bread, nutty | 0.001 | 20.38 | 216.57 | 312.41 |  |
| 90 | 3-methyl-Indole | 1391 | pungent, fecal, indole | 0.00041 | 58.75 | 46.46 | 22.94 |  |
| 91 | dihydro-2-methyl-3(2H)-Furanone | 810 | sweet, buttery, nutty | 0.003 | 51.36 | 26.57 | 21.92 |  |
| 92 | methyl-Pyrazine | 821 | nutty, roasted, chocolate | 0.06 | 1.20 | 28.17 | 70.12 |  |
| 93 | 2-Acetyl-3-methylpyrazine | 1082 | roasted, nutty, flesh | 0.02 | 4.14 | 10.89 | 49.47 |  |
| 94 | 3-ethyl-2,5-dimethyl-Pyrazine | 1081 | roasted, nutty | 0.0086 | 0.71 | 12.00 | 39.24 |  |
| 95 | 2-ethyl-Furan | 703 | nutty, malty, coffee | 0.0023 | 14.28 | 8.58 | 17.15 |  |
| 96 | 2-methyl-3-(methylthio)-Pyrazine | 1184 | roasted, nutty, almond | 0.001 | 9.85 | 3.31 | 4.24 |  |
| 97 | dihydro-5-pentyl-2(3H)-Furanone | 1368 | woody, coconut | 0.0079 | 12.31 | 1.03 | 1.04 |  |
| 98 | 4-Methylthiazole | 818 | nutty, green, tomato | 0.055 | 2.26 | 6.82 | 5.28 |  |
| 99 | 3,5-diethyl-2-methyl-Pyrazine | 1162 | nutty, meaty, vegetable | 0.05 | 0.09 | 1.57 | 8.62 |  |
| 100 | 2,5-dimethyl-Pyrazine | 909 | roasted, nutty, woody | 1.75 | 1.19 | 2.44 | 6.63 |  |
| 101 | 2-methoxy-3-methyl-Pyrazine | 970 | roasted, almond | 0.003 | 3.14 | 1.85 | 4.39 |  |
| 102 | 2-Acetylthiazole | 1020 | nutty, popcorn, roasted | 0.004 | 5.15 | 3.07 | 0.85 |  |
| 103 | tetrahydro-6-methyl-2H-Pyran-2-one | 1095 | fruity, creamy, coconut | 0.02683 | 2.96 | 2.65 | 2.38 |  |
| 104 | 5H-5-Methyl-6,7-dihydrocyclopentapyrazine | 1144 | roasted, malty | 0.05 | 1.88 | 0.89 | 3.19 |  |
| 105 | trimethyl-Pyrazine | 1004 | roasted, nutty | 0.29 | 0.24 | 1.35 | 3.11 |  |
| Terpenoids | | | | | | | | |
| 106 | 2-Methylisoborneol | 1198 | earthy, musty | 0.00048 | 842.97 | 728.06 | 813.45 |  |
| 107 | 2,6,6-trimethyl-1,3-Cyclohexadiene-1-carboxaldehyde | 1386 | spicy, herbal, phenol | 0.003 | 368.15 | 256.07 | 277.30 |  |
| 108 | trans-Rose oxide | 1121 | floral | 0.0005 | 152.62 | 59.77 | 609.50 |  |
| 109 | endo-Borneol | 1170 | woody, pine, camphor | 0.18 | 17.66 | 14.66 | 15.25 |  |
| 110 | Linalool | 1100 | floral, green | 0.006 | 14.41 | 11.63 | 12.04 |  |
| 111 | .beta.-Myrcene | 992 | musty, spicy | 0.015 | 12.21 | 3.38 | 14.44 |  |
| 112 | .beta.-Phellandrene | 1006 | green, herbal | 0.036 | 9.57 | 7.76 | 9.92 |  |
| 113 | Geraniol | 1255 | sweet, floral, fruity | 0.0066 | 11.88 | 4.14 | 3.63 |  |
| 114 | .beta.-Ocimene | 1037 | fruity | 0.034 | 1.08 | 7.79 | 9.45 |  |
| 115 | Carvone | 1242 | fresh, minty | 0.067 | 11.90 | 8.32 | 9.41 |  |
| 116 | Damascenone | 1386 | fruity, sweet | 0.0015 | 3.20 | 3.22 | 8.32 |  |
| 117 | Fenchol | 1117 | malty, woody, sweet | 0.05 | 1.54 | 4.33 | 6.25 |  |
| 118 | Citronellol | 1228 | floral | 0.04 | 0.22 | 1.60 | 7.81 |  |
| 119 | Citronellal | 1154 | waxy, floral, herbal | 0.06 | 3.58 | 2.85 | 3.03 |  |
| 120 | .alpha.-Phellandrene 1 | 1006 | citrus, herbal, green | 0.04 | 0.25 | 1.51 | 5.42 |  |
| 121 | (E)-3,7-dimethyl-2,6-Octadienal | 1270 | citrus, fresh | 0.028 | 1.38 | 1.70 | 2.07 |  |
| Aromatics | | | | | | | | |
| 122 | 1,2-dihydro-1,1,6-trimethyl-Naphthalene | 1354 | green, fresh, licorice | 0.0025 | 1.10 | 21.55 | 39.42 |  |
| 123 | 2-Methoxy-4-vinylphenol | 1316 | spicy, raisin | 0.003 | 14.26 | 1.41 | 3.32 |  |
| 124 | 2-methyl-Naphthalene | 1297 | sweet, floral, woody | 0.004 | 5.74 | 4.17 | 4.32 |  |
| 125 | Naphthalene | 1189 | pungent, dry, tarry | 0.05 | 2.97 | 2.46 | 2.56 |  |
| 126 | Naphthalene, 1,2,3,4-tetrahydro- | 1155 | pungent | 0.05 | 0.78 | 2.77 | 2.83 |  |
| Phenols | | | | | | | | |
| 127 | p-Cresol | 1073 | phenol, animalic, mimosa | 0.00024 | 2172.14 | 988.20 | 2045.26 |  |
| 128 | 3-ethyl-Phenol | 1169 | musty | 0.00085 | 214.35 | 79.32 | 178.14 |  |
| 129 | 2-methyl-Phenol | 1052 | phenol | 0.0039 | 57.82 | 29.14 | 59.92 |  |
| 130 | Phenol | 980 | phenol, medicinal | 0.03 | 33.26 | 27.67 | 26.40 |  |
| Sulfur compounds | | | | | | | | |
| 131 | 3-Methyl-2-butene-1-thiol | 821 | sulfury, onion, smoky | 0.0000002 | 51411.31 | 20292.80 | 21155.87 |  |
| 132 | Diethyl diSulfur | 927 | gassy, onion, garlic | 0.00002 | 9392.82 | 192.82 | 15620.67 |  |
| 133 | Benzenemethanethiol | 1092 | onion, sulfury, garlic | 0.001 | 17.84 | 21.32 | 22.99 |  |
